# Efficacy and safety of pantoprazole and itopride in patients with overlap of gastroesophageal reflux disease and dyspepsia: A prospective, open‐label, single‐arm pilot study

**DOI:** 10.1002/jgh3.12988

**Published:** 2024-02-02

**Authors:** Sundeep Lakhtakia, Aniruddha P Singh, Neeraj Singla, Sana F Memon, Duvvur N Reddy

**Affiliations:** ^1^ Department of Medical Gastroenterology AIG Hospitals Hyderabad India; ^2^ AIG Hospitals Hyderabad India

**Keywords:** GERD symptom assessment scale distress score, itopride, overlapping dyspepsia, pantoprazole, refractory gastroesophageal reflux disease

## Abstract

**Background and Aim:**

Combining proton pump inhibitors (PPIs) with prokinetics can provide synergistic action in patients with gastroesophageal reflux disease (GERD) and overlapping dyspepsia, but data regarding this is lacking.

**Methods:**

This single‐center, prospective study evaluated the efficacy and safety of 6‐week treatment with fixed‐drug combination (FDC) of pantoprazole (PPI) and itopride (prokinetic) in 50 patients with ≥3 month history of GERD and overlapping dyspepsia refractory to pantoprazole. Efficacy was assessed as reduction in GERD symptom assessment scale (GSAS) distress score for 15 symptoms from baseline to week 6. Adverse events (AEs) were monitored up to week 6.

**Results:**

Although heartburn was the most common symptom at week 6 (26.8%), its frequency significantly decreased from baseline (84.0%; *P* <0.01). A similar trend was observed for other symptoms: pressure/discomfort inside chest (19.5%), belching (14.6%), regurgitation (12.2%), bloating (9.8%), flatulence (9.8%), early satiety (7.3%), acidic/sour taste in mouth (7.3%), nausea (7.3%), frequent gurgling in stomach/belly (4.9%), and pressure/lump in throat (2.4%). Mean distress scores of all symptoms markedly decreased at week 6. Three AEs (*n* = 2) of moderate intensity were reported.

**Conclusion:**

The FDC of pantoprazole and itopride showed favorable efficacy and safety in patients with GERD and overlapping dyspepsia refractory to pantoprazole monotherapy. Nevertheless, further studies are warranted.

## Graphical abstract

The main objective of this study was to evaluate the efficacy and safety of a fixed‐dose combination (FDC) of pantoprazole (PPI) and itopride (prokinetic) in the treatment of GERD patients with overlapping symptoms of dyspepsia. Our research showed that heartburn was the most frequently experienced symptom at baseline (84%), and a drastic change from baseline was observed at week 6 in its frequency in patients experiencing it (26.8%, *P* <0.001), highlighting the FDC's favorable efficacy and safety in these patients.

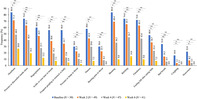

## Introduction

Gastroesophageal reflux disease (GERD) is a usual condition that develops when the gastric content refluxes into the mouth or hypopharynx and causes troublesome symptoms with or without visible damage to the esophageal mucosa.^1^ The most common symptoms of GERD include heartburn, regurgitation, dysphagia, and chest pain.^2^, ^3^ Dyspepsia and GERD are correlated because both can occur simultaneously in an individual. Dyspepsia is a syndrome characterized by postprandial fullness, early satiety, epigastric pain and burning, bloating in the upper abdomen, nausea, vomiting, and belching.^4^ In India, the prevalence of GERD is reported to be 7.6–30%, demonstrating that the magnitude of GERD is in fact comparable to that reported in the Western countries.^5^ It is estimated that up to 41.5% of GERD patients have symptoms of dyspepsia.^6^ An overlap between GERD and non‐diagnosed dyspepsia was found in 50% of GERD patients.^7^

Acid suppression is the main mechanism of action for treating GERD^8^ Proton pump inhibitors (PPIs) increase the intragastric pH and suppress gastric acid by inactivating gastric proton pumps responsible for acid secretion.^9^ Long‐term PPI therapy with pantoprazole is useful in patients with chronic or complicated GERD, with no major safety issues, but it does not help in improving the underlying disturbance in gut motility or the tone of the lower esophageal sphincter.^8^, ^10^ Several studies have shown that up to 40% of patients with GERD report either partial or complete lack of response to a standard PPI dose once daily.^11^

PPI‐refractory GERD is defined as persistent and troublesome GERD symptoms nonresponsive to at least 8 weeks of a standard dose of PPI.^12^ Refractory GERD cases require more aggressive acid‐suppressive therapy for effective management of acid reflux. Currently, physicians prescribe either a double dose of PPI or a combination of PPIs and prokinetic agents for severe and resistant GERD. The combination of PPI and prokinetic agents has been found to be more effective in acid reflux management in comparison to PPIs alone.^13^, ^14^, ^15^ Prokinetics, when combined with a PPI, cause a significant increase in the bioavailability of PPI, thereby eliciting a favorable effect of PPI therapy.^16^

However, there is disagreement regarding the efficacy and safety of combined prokinetic and PPI therapy in GERD.^17^, ^18^ In light of limited data on the combination of PPIs and prokinetics in GERD with overlapping dyspepsia, the present pilot study evaluated the efficacy and safety of a fixed‐dose combination (FDC) of pantoprazole 40 mg and itopride 150 mg in the treatment of GERD patients with overlapping symptoms of dyspepsia.

## Methods

### Study design

Eligible patients with GERD and overlapping symptoms of dyspepsia not adequately responding to pantoprazole 40 mg alone were screened to enroll a total of 50 patients. The total duration of patient participation in the study was 6 weeks, and the total duration of study completion including data analysis was approximately 11 months. After written informed consent was obtained, patients received the study drug, that is, pantoprazole 40 mg + itopride 150 mg (Ganaton Total by Abbott India Ltd.) once daily. The study protocol was reviewed and approved by the institutional review board (IRB). The study was registered with the Clinical Trials Registry of India on 17 September 2020 (CTRI/2020/09/027876).

### Eligibility criteria

Patients aged ≥18 years who had symptoms of GERD (i.e., heartburn and/or regurgitation at least twice a week for ≥3 months) along with overlapping symptoms of dyspepsia (postprandial fullness, early satiety, epigastric pain, and burning for ≥3 days a week for the last 3 months) as per ROME IV criteria,^4^ and who were refractory to pantoprazole 40 mg alone for ≥8 weeks were included in the study. Additionally, patients were required to have discontinued, at least 1 week before enrolment, medications affecting esophageal motility, including but not limited to prokinetics, PPIs, H2 receptor antagonists, and cholinergic and/or anti‐cholinergic agents.

Key exclusion criteria were patients with Barrett's esophagus, esophageal stricture, or active gastroduodenal ulcer; patients with a history of gastric, duodenal, or esophageal surgery, any active malignancy, or heart failure and/or clinically significant renal disease (serum creatinine >1.5 mg/dL); and patients with a known history of liver disease, hypotension, atrophic gastritis, *Clostridium difficile*‐related colitis, interstitial nephritis, osteoporosis, vitamin B12 deficiency, or hypomagnesemia.

### Study endpoints

The primary endpoint was efficacy as assessed by a reduction in the GSAS distress score from baseline to weeks 2, 4, and 6. Secondary endpoints were (i) frequency of heartburn, regurgitation, or dyspepsia symptoms in the last 7 days on GSAS, and (ii) change in total daily duration of heartburn, regurgitation, or dyspepsia over the last 7 days, from baseline to weeks 2, 4, and 6. Safety endpoints included incidence of adverse events (AEs) and serious adverse events (SAEs), and global tolerability assessment by the patients and the investigator at weeks 2, 4, and 6.

### Study assessments

#### GSAS distress scoring

GSAS is a self‐administered, patient‐oriented questionnaire to evaluate the frequency, severity, and degree of distress for the following 15 specific symptoms: heartburn, pressure or discomfort inside chest, regurgitation, acidic or sour taste in mouth, frequent gurgling in stomach or belly, pressure or lump in throat, nausea, burning pain in throat, bloating, belching, flatulence, feeling full after eating little, bad breath, coughing, and hoarseness.^19^, ^20^ GSAS distress scores were assigned based on the presence of these symptoms and their distress ratings. The degree of distress for any symptom was rated on a 4‐point scale: score 0 = not at all, 1 = somewhat, 2 = quite a bit, or 3 = very much. The total distress score was calculated by summing scores across symptoms and dividing by the total number of non‐missing symptom scores. The GSAS distress score was computed if 12 or more symptoms were scored. Patients with four or more missing symptom scores were considered as having missing GSAS distress scores.

#### Safety and tolerability assessments

AEs were collected and evaluated for their relatedness to the study drug, severity, seriousness, and outcome. Global tolerability assessment by patients and investigator was performed independently at weeks 2, 4, and 6 using a 3‐point scale with scores 0, 1, and 2 representing good, moderate, and poor tolerability, respectively.

### Statistical analysis

Descriptive and summary statistics were calculated to detect improvement on functional and clinical tests and GSAS distress score from baseline to weeks 2, 4, and 6. Statistical analyses were performed at α = 0.05 significance level. The primary and secondary outcome measures along with safety outcomes of the study score were summarized. For continuous data, mean and standard deviation were calculated, and for categorical data, the number and percentage of subjects within each category were reported. Student's paired *t*‐test and McNemar's test were used to compare significant change between the continuous variables and change in frequencies from baseline to weeks 2, 4 and 6, respectively. All statistical processing was performed using the IBM SPSS Statistics for Windows, Version 20.0 (IBM Corp. Armonk, NY; released 2011). Because this was a pilot study, no formal sample size calculations were performed, but a sample size of 50 patients was thought to be adequate to meet the study objective.

## Results

### Patient demographics

**Figure 1:**
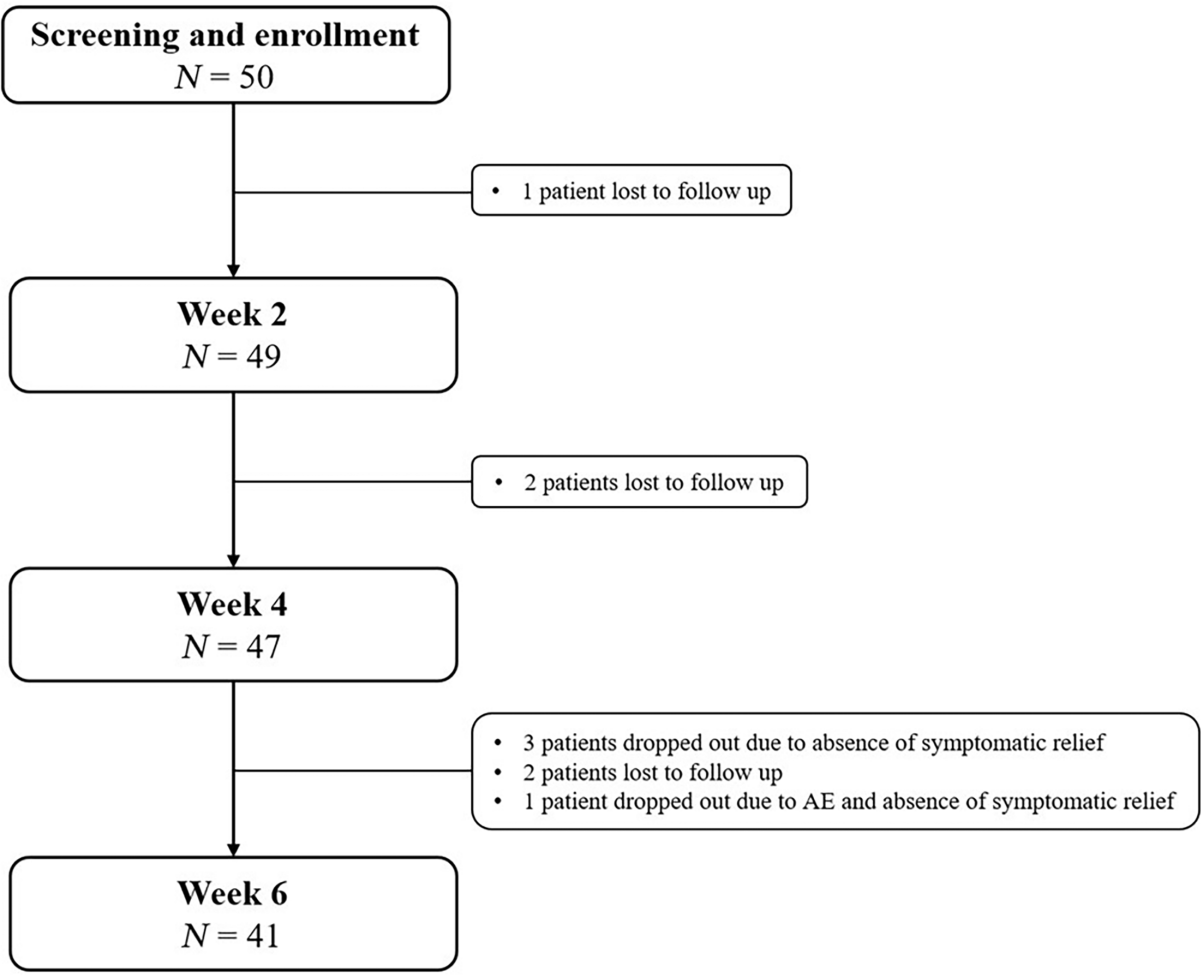
Patient disposition. AE, adverse event.

In all, 50 patients participated in this study. At week 2 one patient, at week 4 two patients, and at week 6 two more patients were lost to follow‐up. Three patients dropped out because of absence of symptomatic relief and one patient because of AE and no symptomatic relief (Fig. 1). Thus, at weeks 2, 4, and 6, analyses were carried out with 49, 47, and 41 patients, respectively.

Table 1 shows the baseline characteristics of the study participants. The study population consisted of 84.0% males, and the mean age was 44.0 (12.26) years (range 21–69 years). The mean BMI was 24.9 (4.24) kg/m^2^. In all, 22% patients had a history of alcohol consumption and 14% had a history of smoking. With regard to medical history, 20% of patients had endocrine and metabolic diseases, while 8% were suffering from cardiovascular diseases in the study cohort.

**Table 1:** Baseline characteristics

Parameters	Overall (*N* = 50)
Age (years), mean (SD)	44.0 (12.26)
Height (cm), mean (SD)	167.2 (7.26)
Weight (kg), mean (SD)	70.5 (12.58)
BMI (kg/m^2^), mean (SD)	24.9 (4.24)
Male gender, *n* (%)	42 (84.0)
History of alcohol use, *n* (%)	11 (22.0)
History of smoking, *n* (%)	7 (14.0)
Medical history, *n* (%)	
Endocrine and metabolic diseases	10 (20.0)
Cardiovascular diseases	4 (8.0)

BMI, body mass index; SD, standard deviation.

### Efficacy of FDC

#### Change in GSAS distress scores following 6 weeks of treatment

Change in GSAS overall and symptom‐wise distress scores were assessed at weeks 2, 4, and 6 (Table 2). Among 49 evaluable patients at week 2, treatment with the FDC significantly reduced the overall GSAS distress score by 0.9 (0.70) from a baseline score of 2.4 (0.37) (*P* < 0.01). Similarly, among 47 evaluable patients at week 4, the overall score decreased by 1.4 (0.81; *P* < 0.001), and among 41 evaluable patients at week 6, it decreased by 2.0 (0.61; *P* < 0.001).

**Table 2:** Change in GSAS distress scores from baseline up to week 6

GSAS symptoms	Baseline (*N* = 50)		Week 2 (*N* = 49)				Week 4 (*N* = 47)				Week 6 (*N* = 41)			
Distress score (mean [SD])	*n*	Absolute	*n*	Absolute	Change	*P‐*value	*n*	Absolute	Change	*P‐*value	*n*	Absolute	Change	*P‐*value
Overall score	50	2.6 (0.48)	49	1.5 (0.67)	0.9 (0.70)	<0.01	47	1.0 (0.76)	1.4 (0.81)	<0.01	41	0.4 (0.50)	2.0 (0.61)	<0.01
Heartburn	42	2.6 (0.48)	35	1.8 (0.77)	0.8 (0.68)	<0.01	23	1.5 (0.79)	1.2 (0.74)	<0.01	11	1.1 (0.30)	1.5 (0.52)	<0.01
Feeling of pressure or discomfort inside chest	37	2.6 (0.50)	31	1.7 (0.69)	0.8 (0.64)	<0.01	19	1.5 (0.70)	1.2 (0.69)	<0.01	8	1.0 (0.00)	1.5 (0.53)	<0.01
Regurgitation	30	2.4 (0.63)	23	1.8 (0.74)	0.6 (0.78)	<0.01	13	1.5 (0.78)	1 (0.91)	<0.01	5	0.8 (0.45)	1.8 (0.45)	<0.01
Acidic or sour taste in mouth	28	2.5 (0.51)	19	1.7 (0.73)	0.8 (0.71)	<0.01	9	1.6 (0.88)	1.1 (0.78)	<0.01	3	1.0 (0.00)	1.7 (0.58)	0.04
Frequent gurgling in stomach or belly	28	2.3 (0.71)	17	1.6 (0.71)	0.8 (0.73)	<0.01	6	1.8 (0.75)	0.7 (0.52)	0.03	2	1.5 (0.71)	1.5 (0.71)	0.205
Feeling of pressure or lump in throat	15	2.1 (0.70)	11	1.8 (0.75)	0.6 (0.52)	0.01	5	2.0 (0.71)	0.6 (0.55)	0.07	1	1.0 (0.00)	‐	‐
Nausea	29	2.2 (0.58)	21	1.7 (0.64)	0.6 (0.59)	<0.01	14	1.4 (0.50)	1 (0.74)	<0.01	3	0.7 (0.58)	1.7 (0.58)	0.04
Burning pain in throat	15	2.0 (0.59)	11	1.5 (0.52)	0.5 (0.52)	0.02	4	2.0 (0.0)	0.3 (0.5)	0.39	‐	0.0 (0.00)	‐	‐
Bloating	42	2.6 (0.59)	34	1.6 (0.70)	1.0 (0.75)	<0.01	17	1.5 (0.72)	1.4 (0.79)	<0.01	4	1.0 (0.00)	1.8 (0.50)	0.01
Belching	37	2.7 (0.53)	32	1.8 (0.72)	1.0 (0.65)	<0.01	21	1.5 (0.68)	1.4 (0.74)	<0.01	6	0.7 (0.52)	2.2 (0.75)	0.001
Flatulence	36	2.5 (0.56)	31	1.6 (0.62)	1.0 (0.72)	<0.01	20	1.5 (0.60)	1.2 (0.96)	<0.01	4	1.0 (0.80)	1.8 (1.26)	0.07
Feeling full after eating little	24	2.5 (0.51)	18	1.8 (0.79)	0.7 (0.67)	<0.01	11	1.5 (0.69)	1.3 (0.67)	<0.01	3	1.3 (0.58)	1.7 (0.58)	0.04
Bad breath	17	1.7 (0.49)	7	1.4 (0.53)	0.4 (0.53)	0.08	3	1.3 (0.58)	0.3 (0.58)	0.42	‐	0.0 (0.00)	‐	‐
Coughing	8	1.5 (0.53)	2	1.5 (0.71)	0.5 (0.71)	0.50	1	2.0 (0.00)	‐	‐	‐	0.0 (0.00)	‐	‐
Hoarseness	11	1.6 (0.67)	4	1.3 (0.50)	0.8 (0.50)	0.06	2	1.0 (0.00)	1 (1.41)	0.50	‐	0.0 (0.00)	‐	‐

*P*‐value by paired *t*‐test.

DS, distress score.

**Figure 2:**
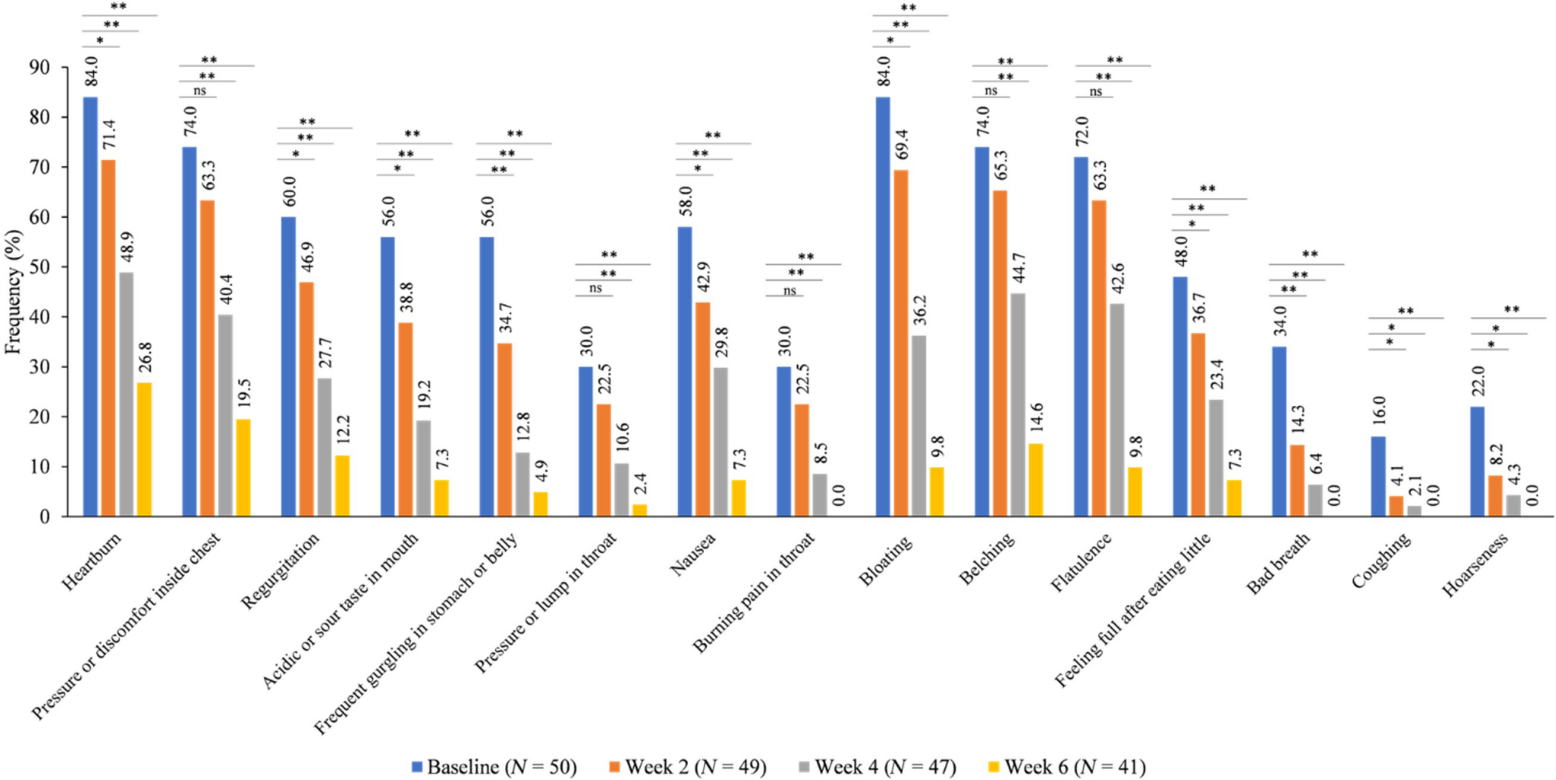
Frequency of GSAS symptoms from baseline to week 6. *P < 0.05 and **P < 0.01 by McNemar test. GERD, gastroesophageal reflux disease; GSAS, GERD symptom assessment scale.

With regard symptom‐wise scores, heartburn and bloating were the most frequently reported symptoms by the participants at baseline (Fig. 2). In all, 42 out of 50 patients (84.0%) reported heartburn and bloating, which was followed by a feeling of pressure or discomfort inside the chest (37/50; 74.0%), belching (37/50; 74.0%), flatulence (36/50; 72.0%), food coming back to mouth (30/50; 60.0%), nausea (29/50; 58.0%), acidic or sour taste in mouth (28/50; 56.0%), and gurgling (28/50; 56.0%). At week 2, there was a decrease in the mean distress scores for all symptoms. Distress scores of symptoms such as heartburn or a burning pain inside chest, regurgitation, acidic or sour taste in mouth, gurgling, nausea, bloating, feeling full after eating little, bad breath, coughing, and hoarseness showed statistically significant change from baseline (*P* < 0.05). Heartburn was experienced by 71.4% of patients, bloating by 69.4%, belching by 65.3%, and flatulence and pressure or discomfort inside chest by 63.3%. Overall, the mean distress score for every symptom at week 2 was found to be decreased compared to baseline. At week 4, heartburn continued to be the most frequent symptom (48.9%), followed by belching (44.7%), flatulence (42.6%), pressure or discomfort inside chest (40.4%), bloating (36.2%), nausea (29.8%), and regurgitation (27.7%). At week 6, although heartburn was the most frequently experienced symptom (26.8%), there was a drastic change from baseline in its frequency in patients experiencing it (84.0%; *P* <0.001). Other symptoms also showed a similar trend. Moreover, there was a marked decrease in the mean distress scores of all symptoms at 6 weeks (Table 2).

#### Change in frequency and duration of gastrointestinal symptoms in the past 7 days over the study duration

The mean (SD) number of GI symptoms (i.e., heartburn, regurgitation, and dyspepsia experienced at baseline) was 8.0 (1.77) per patient, which declined to 6.0 (2.93) at week 2; 3.6 (3.15) at week 4; and 1.2 (1.92) at the end of the study (week 6) (Table 3). The mean (SD) frequency of GI symptoms at baseline was 59.7 (10.25) per patient, which reduced to 41.0 (18.84) at week 2; 27.1 (20.22) at week 4; and 10.7 (14.11) at week 6. The mean (SD) of total daily duration of heartburn, regurgitation, or dyspepsia in the past 7 days declined from 79.3 (10.8) hours at baseline to 7.6 (18.7), 1.6 (3.21), and 0.0 (0.0) hours at weeks 2, 4, and 6, respectively.

**Table 3:** Frequency, distress scores, and duration of gastrointestinal symptoms over the last 7 days at baseline and weeks 2, 4, and 6

Parameter, mean (SD)	Baseline (*N* = 50)	Week 2 (*N* = 49)	Change from BL	*P‐*value	Week 4 (*N* = 47)	Change from BL	*P‐*value	Week 6 (*N* = 41)	Change from BL	*P‐*value
Average number of GI symptoms*	8.0 (1.77)	6.0 (2.93)	1.9 (2.54)	<0.01	3.6 (3.15)	4.4 (3.28)	<0.01	1.2 (1.92)	6.7 (2.30)	<0.01
Average frequency of all symptoms	59.7 (10.25)	41.0 (18.84)			27.1 (20.22)			10.7 (14.11)		
Average total daily duration (hour) in past 7 days	79.3 (10.78)	7.6 (18.74)*	68.7 (22.96)	<0.01	1.6 (3.21)*	74.7 (15.57)	<0.01	0.0 (0.00)*	76.3 (12.75)	<0.01

* Based on data available for 7 patients at week 2; 11 patients at week 4; and 19 patients at week 6.

GI symptoms include heartburn, regurgitation, or dyspepsia.

BL, baseline; GI, gastrointestinal; SD, standard deviation.

### Safety and tolerability of FDC

Three AEs were experienced by two (4.3%) patients at week 4. These events were stomach upset, stomach pain, and stomach and epigastric pain; none of these events was serious or severe in nature and all were resolved. No other AEs or SAEs were reported during the study period.

Tolerability of FDC was assessed independently by patients and the investigator for each patient at weeks 2, 4, and 6. Overall, the patient and investigator assessments indicated good tolerability of the FDC at week 2 (*n* = 48, 98.0%), week 4 (*n* = 46, 97.9%), and week 6 (*n* = 41, 100%). Tolerability was rated as moderate by one patient at week 2 (2.0%) and as poor by one patient at week 4 (2.1%); both these findings were also corroborated by the investigator.

## Discussion

This prospective observational pilot study included 50 GERD patients with overlapping symptoms of dyspepsia who were refractory to pantoprazole 40 mg alone for at least 8 weeks. Findings from the present study suggest that FDC of pantoprazole 40 mg and itopride 150 mg was efficacious in reducing GSAS distress scores from baseline to weeks 2, 4, and 6 in patients with GERD and overlapping symptoms of dyspepsia. In a double‐blind clinical trial, 60 dyspeptic patients with heartburn and/or regurgitation given either omeprazole plus domperidone or omeprazole alone for 2 weeks showed statistically significant mean improvement in frequency scale for the symptoms of GERD score in the omeprazole plus domperidone group (*P* = 0.02), indicating that the combination of a PPI and a prokinetic provides better results than with PPI alone.^14^

Jung *et al*., in a meta‐analysis of 16 studies involving 719 participants receiving PPI + prokinetics and 727 receiving PPI monotherapy, concluded that PPI + prokinetics treatment resulted in a significant reduction in the global symptoms of GERD irrespective of the prokinetic type, refractoriness, and ethnicity. At least 4 weeks of PPI + prokinetics treatment was found to be more beneficial than PPI monotherapy with respect to global symptom improvement. However, the quality‐of‐life (QoL) scores were not improved with PPI + prokinetics treatment. AEs observed were similar in both groups.^21^

Itopride is a commonly used dopamine (D2) receptor antagonist for the treatment of functional dyspepsia (FD). In addition to blocking the D2 receptor, it inhibits acetylcholinesterase, the key neurotransmitter involved in regulating gastric contractility. Increases in acetylcholine levels promote gastric contractility, enhance lower esophageal sphincter pressure, and accelerate gastric emptying.^22^

A randomized study evaluating the clinical efficacy and safety of dual therapy of pantoprazole and itopride (pantoprazole 40 mg twice daily and itopride 50 mg thrice daily for 4 weeks) *versus* pantoprazole alone (pantoprazole 40 mg twice daily for 4 weeks) found significantly higher symptom relief with the former in patients with GERD (74.5% *vs* 62.5%, *P* < 0.001) and also improved QoL.^17^

Kim *et al*. randomized 26 patients with GERD to receive 150 or 300 mg itopride thrice daily for 1 month and found significant improvement in all symptoms including heartburn in both the groups.^23^

A recent study evaluating itopride using validated, patient‐reported outcome measures suggested the efficacy of itopride especially in patients with FD.^24^ Sawant *et al*. compared the efficacy of itopride and domperidone in non‐ulcer dyspepsia and reported moderate to complete symptom relief in 81% of the patients following itopride treatment compared to 70% receiving domperidone.^25^ In another open‐label, non‐comparative study by Shenoy *et al*. on the efficacy and tolerability of itopride hydrochloride in patients with non‐ulcer dyspepsia, moderate to complete relief of symptoms was reported by 73% of patients at 2 weeks while 17% reported only slight improvement.^26^

Four of six trials on the role of itopride reported a significant improvement in FD symptoms after 2–8 weeks of treatment,^24^, ^25^ whereas two trials found no improvement compared to placebo.^27^, ^28^

Beneficial effects of itopride were seen in a large‐scale, placebo‐controlled, phase IIb trial involving FD patients, but these benefits were not observed in another trial. This trial was similar in study design, with similar weeks of double‐blind treatment, but had excluded *Helicobacter pylori*‐positive patients and those with mild symptoms, thereby explaining the differences between the studies.^28^, ^29^

A meta‐analysis of nine randomized, placebo‐controlled trials involving 2620 individuals with FD was performed, where 1372 received itopride and 1248 (control group) were treated with drugs such as domperidone, mosapride, or placebo. Patients in the itopride group showed better results in terms of global patient assessment, postprandial fullness, and early satiety with lower side effects.^22^

Venkatesh *et al*., in their 3‐week‐long, open‐label, multicenter study of 743 patients with diabetic gastroparesis, evaluated the efficacy of pantoprazole and itopride. The efficacy parameters were nausea, vomiting, feeling full after eating little, bloating, postprandial fullness, epigastric pain, and regurgitation. The severity and frequency of all the symptoms were found to be significantly improved (*P* < 0.001).^30^ Another placebo‐controlled, randomized, crossover trial showed accelerated emptying of both solids and liquids in the itopride group compared with the placebo group in patients with long‐standing diabetes.^31^

From the safety perspective, the FDC of pantoprazole and itopride was found to be safe, with a total of three AEs reported over a span of 6 weeks. These events included epigastric pain or gastrointestinal upset, which were self‐limiting. Around 98%, 97.9%, and 100% of patients at weeks 2, 4, and 6, respectively, rated that the FDC of pantoprazole 40 mg and itopride 150 mg had good tolerability as per the global tolerability assessment scale. A similar trend was observed for physician‐reported tolerability.

The safety of itopride has been established in multiple studies so far. Itopride is not associated with any AEs of the central nervous system, as it does not cross the blood–brain barrier because of high polarity, making it a safer drug compared to metoclopramide.^32^, ^33^ Previous studies on the use of itopride have shown AE rate ranging from 1.5% to 7%, which is comparable to our study.^25^, ^26^, ^27^, ^28^, ^34^ Commonly reported AEs with itopride include mild elevation in prolactin levels, abdominal pain, diarrhea, constipation, and nausea. No reports of itopride‐induced prolonged Q–T intervals were found.^27^ Therefore, itopride can be considered a safe treatment option for patients with GERD and FD.

This study had a few limitations. Being a pilot study, the study population was small, and consecutive eligible patients were included. Moreover, planned follow‐up visits at the site were not possible due to the COVID‐19 pandemic and so complete follow‐up data could not be registered. The efficacy and safety of pantoprazole and itopride need to be assessed in a larger population group with GERD and overlapping symptoms of dyspepsia and in the presence of a control group. The study results cannot be generalized because the follow‐up interval was too short to comment about any sustainable effects. Factors such as *H. pylori* status or the coexistence of any ulcer dyspepsia were not considered, as none of the patients underwent endoscopy.

## Conclusion

In conclusion, the FDC of pantoprazole 40 mg and itopride 150 mg exhibited a good safety profile and was effective in patients with symptoms of dyspepsia with GERD overlap. Significant reduction in symptoms such as heartburn, regurgitation, or dyspepsia with use of this drug combination was observed over a period of 6 weeks. However, further large randomized controlled trials are warranted to establish the efficacy of this FDC in patients with overlapping dyspepsia and GERD.
